# Vaccines for Improved Cellular Immunity to Influenza

**DOI:** 10.1016/j.ebiom.2018.03.001

**Published:** 2018-03-05

**Authors:** Graham Pawelec, Janet McElhaney

**Affiliations:** a Second Department of Internal Medicine, University of Tübingen, Tübingen, Germany; b Health Sciences North Research Institute, Sudbury, ON, Canada; c John van Geest Cancer Research Centre, Nottingham Trent University, Nottingham, UK

Influenza disease remains a major global public health problem, especially for the elderly, despite intensive efforts to develop more effective vaccines. The seasonal vaccines currently in use suffer from many disadvantages, particularly their poor capacity to elicit cellular immunity and the strain specificity of the antibody response. The selection of target strains is based on prediction of dominant emerging strains in the upcoming season, which may not be altogether accurate. Many efforts in the search for a “universal” influenza vaccine have been directed towards eliciting antibodies binding to the stem region of the viral hemagglutinin conserved between strains, rather than the highly variable globular head domain (Ekiert and Wilson, 2012). However, there is a great deal of evidence suggesting that optimal protection against clinical disease correlates more closely with the ability of the vaccine to stimulate T cell responses than with the antibody titers that are routinely assessed as a surrogate for vaccine efficacy (McElhaney et al., 2006). Indeed, this is underlined by findings from epidemiological studies showing that the presence of pre-challenge T cells responsive to influenza A nucleoprotein (NP) correlated with some protection against infection, even in the absence of antibodies (Hayward et al., 2015). An ideal vaccine would therefore be an “off-the-shelf” product capable of eliciting long-lasting protective T-cell and B cell-mediated immunity independent of the particular viral strains in circulation at any one time, and which is equally effective in at-risk populations, such as the elderly or diabetics (Remschmidt et al., 2015). The *EBioMedicine* paper by Coughlan et al. (2018--in this issue) describes significant steps taken in this direction in a phase I clinical trial of vaccines designed to boost such T cell responses in both younger and older adults. Testing new vaccines in the elderly is extremely important because older people commonly respond poorly to current vaccines, most likely primarily due to deficits in antigen presentation by dendritic cells and T cell responses rather than any intrinsic inability of their B cells to produce antibodies (van Duin et al., 2007). Building on long-standing earlier investigations (Lillie et al., 2012), Coughlan et al. (2018--in this issue) determined the safety of heterologous vaccination with the same matrix protein-1 (M1) and NP moieties delivered by two different viral vectors in younger and older people. Vaccine safety was assessed and stimulation of T cell responses measured by interferon-γ ELISPOT. Encouragingly, both vaccines were well-tolerated and when used sequentially 8 weeks apart significantly increased M1- and NP-stimulated T cell responses in vitro; importantly, responses were maintained long-term, at least in younger people (>18 months), a significant advance over the natural retention of T cell responses after infection, which tend to wane quite rapidly (Hayward et al., 2015). Similar responses were seen in the elderly but unfortunately they were not followed up beyond 6 months. Thus it remains undetermined whether responses would still be enhanced at later times in the elderly as well as the young, which is likely to be important for protection. Nonetheless, the evidence is clear that as with other antigens, the heterologous prime-boost strategy reported here for the first time by Coughlan et al. (2018--in this issue) using two different viral vectors delivering the same immunogens succeeded in enhancing T cell-mediated immunity in both younger and older people. It will be important to determine whether there are sex differences in the response to these vaccines, but the current study was too small to approach this question. Many other variables remain to be explored, especially when determining correlates of protection in older adults which must take the effects of frailty into account. It will also be important to establish whether the Cytomegalovirus serostatus of the vaccinees or other factors such as diabetes influence their response, because studies have shown that these variables may not affect antibody responses but can impact markedly on T cell responses (Haq et al., 2017). However, no data on CMV serostatus, diabetes, use of beta-blockers (Pawelec, 2017) etc. are available in the present paper, but these are details that can be accounted for in larger-scale trials.

Although a major limitation recognized by the authors is the lack of documentation of any clinical protection in this small study, it is argued that their immune monitoring of T cell responses following vaccination suggests that efficacy should be enhanced. This assertion was made on the basis of a previous small challenge study in which protection was directly correlated with the T cell measures tested in the present study (Lillie et al., 2012). The pilot data presented in Coughlan et al. (2018--in this issue) clearly warrant larger trials assessing efficacy of clinical protection in the real world, especially in the elderly, and close monitoring of the immune responses enhanced by the vaccines.

## Disclosure

The authors declared no conflicts of interest.
